# Current Applications and Immunological Considerations of *Salmonella enterica* Serovar Typhimurium as a Vaccine Vector

**DOI:** 10.3390/microorganisms14020492

**Published:** 2026-02-18

**Authors:** Adam S. Hassan, Kaitlin Winter, Charles M. Dozois, Brian J. Ward, Momar Ndao

**Affiliations:** 1 Faculty of Medicine and Health Sciences, McGill University, Montreal, QC H3G 2M1, Canada; adam.hassan@mail.mcgill.ca; 2 Vaccine Evaluation Center, BC Children’s Hospital Research Institute, Vancouver, BC V5Z 4H4, Canada; kaitlin.winter@bcchr.ca; 3 Department of Pediatrics, University of British Columbia, Vancouver, BC V6H 3V4, Canada; 4 Centre Armand-Frappier Santé Biotechnologie, Institut National de Recherche Scientifique, Laval, QC H7V 1B7, Canada; charles.dozois@inrs.ca; 5 Infectious Diseases and Immunity in Global Health Program, Research Institute of the McGill University Health Centre, Montreal, QC H4A 3J1, Canada; brian.ward@mcgill.ca; 6 Department of Microbiology and Immunology, McGill University, Montreal, QC H3A 2B4, Canada; 7 Division of Experimental Medicine, McGill University, Montreal, QC H4A 3J1, Canada; 8 National Reference Centre for Parasitology, Research Institute of the McGill University Health Centre, Montreal, QC H4A 3J1, Canada

**Keywords:** *Salmonella enterica* serovar Typhimurium, live attenuated vaccines, vaccine vectors, mucosal immunity, host–pathogen interactions

## Abstract

Live attenuated *Salmonella enterica* serovar Typhimurium has been investigated for decades as an orally delivered vaccine vector due to its ability to target the intestinal mucosa and engage both innate and adaptive immune responses. In humans, *S.* Typhimurium infection is largely restricted to the gastrointestinal tract, distinguishing it from *Salmonella Typhi* and providing a rationale for its use in mucosal vaccine strategies. In this review, we discuss the biological features of *S.* Typhimurium that support its use as a vaccine vector and summarize current understanding of the immune responses generated during wild-type infection, including innate activation and downstream T cell and B cell responses. We compare key biological differences between *Salmonella* Typhi and *S*. Typhimurium and outline emerging vector design strategies, including delayed attenuation and chromosomal integration of heterologous antigens. We then review applications of attenuated *S.* Typhimurium vectors targeting viral, bacterial, and parasitic pathogens, highlighting shared immunological outcomes and design principles across platforms. Finally, we discuss recent advances in vector engineering, including chromosomal integration of heterologous antigens, as well as remaining gaps in knowledge related to the durability of immune responses and translational considerations.

## 1. Introduction

Vaccines have been remarkably effective in controlling and, in some cases, eliminating infectious diseases. Early vaccine strategies relied on exposure to attenuated pathogens to prevent severe outcomes following wild-type infection. Despite substantial advances in vaccine technology, live attenuated vaccines remain among the most effective platforms and have been successfully used to prevent diseases such as smallpox, poliomyelitis, measles, mumps, and rubella [1]. Attenuated and inactivated bacterial vaccines have also been developed with varying degrees of success, including those targeting cholera, tuberculosis, and typhoid fever [2,3,4].

*Salmonella* species have been studied for decades as candidates for orally delivered live attenuated vaccines and as vectors for heterologous antigen delivery [5]. The live attenuated *Salmonella enterica* Typhi strain Ty21a is administered orally for the prevention of typhoid fever and, using a four-dose regimen, provides protection for up to five years [6]. Although Ty21a has been extensively evaluated as a vector for the delivery of foreign antigens, these efforts have met with limited success. While Ty21a possesses several attractive features as a vaccine platform, its modest efficacy as a vector has raised questions regarding the broader utility of typhoidal *Salmonella* strains for heterologous antigen delivery.

In contrast, *Salmonella enterica* serovar Typhimurium is generally restricted to the intestinal mucosa in humans and induces strong mucosal and cellular immune responses. These properties make non-typhoidal *Salmonella* strains particularly attractive candidates for the development of mucosal vaccine vectors. Advances in attenuation strategies, molecular genetics, and antigen expression systems have renewed interest in repurposing attenuated *S.* Typhimurium strains for vaccine applications. In parallel, an improved understanding of mucosal immunology has provided new insights into how *Salmonella*-based vectors may be leveraged to induce protective immune responses against a wide range of pathogens.

Recent reviews have highlighted the expanding use of *Salmonella*-vectored vaccines in veterinary and agricultural settings, underscoring the versatility of this platform across host species and disease targets [7]. However, comparatively less emphasis has been placed on synthesizing the immunological mechanisms, design principles, and translational considerations relevant to human-targeted, mucosal *Salmonella*-vectored vaccines.

In this review, we summarize the biology of *Salmonella* Typhimurium relevant to vaccine vector design, outline the innate and adaptive immune responses elicited during infection, and discuss current applications of live attenuated *Salmonella*-vectored vaccines targeting viral, bacterial, and parasitic pathogens. We also examine key considerations for platform development, including safety, antigen expression strategies, and translational challenges. Together, this work aims to provide a framework for understanding the potential and limitations of attenuated non-typhoidal *Salmonella* as vaccine vectors.

## 2. *Salmonella* Biology

*Salmonella* spp. are Gram-negative, facultatively anaerobic bacteria and intracellular pathogens of the Enterobacteriaceae family. Though closely related to *Escherichia coli*, all species of *Salmonella* are considered pathogenic [8]. The genus *Salmonella* comprises two species, *S. bongori* and *S. enterica*, where the former includes 22 serovars and the latter contains over 2500 serovars [9]. Gastrointestinal and extraintestinal pathovars of vertebrate hosts make up subspecies of *S. enterica*, including *S.* Typhi and *S.* Typhimurium.

*S.* Typhi is an oral pathogen that crosses the intestinal mucosa and disseminates systemically in humans [10]. In contrast, *S.* Typhimurium exhibits a distinct pathology in humans and is generally restricted to the small intestine and cecum, where it elicits robust mucosal immune responses. Although *S.* Typhimurium is commonly used as a murine model for *S.* Typhi infection, important differences in host specificity and disease manifestation should be considered when extrapolating between species.

Following oral infection, the majority of the bacterial population resides in the intestinal lumen; however, a subset of bacteria infects intestinal epithelial cells (IECs) [11,12,13]. *S.* Typhimurium preferentially targets M cells within Peyer’s patches and can alter epithelial tight junctions to facilitate transepithelial migration [14]. *S.* Typhimurium induces host cell macropinocytosis, allowing bacterial uptake.

Once internalized, *S.* Typhimurium typically resides within a modified phagosome known as the Salmonella-containing vacuole (SCV). This bacterial maintained vacuole is critical for intracellular survival and replication. In some strains, bacteria can escape the SCV and undergo hyper-replication within the host cell cytosol, contributing to dissemination and host cell damage [15].

## 3. Attenuating *Salmonella* Typhimurium

Several strategies have been developed to attenuate *Salmonella*, many of which have been comprehensively reviewed elsewhere. In particular, reviews by Galen et al. and Curtiss et al. describe attenuated strains evaluated in clinical trials and outline the genetic strategies underlying attenuation in detail [16,17]. These approaches are therefore summarized only briefly here.

Early attenuation strategies relied on chemical mutagenesis to introduce random mutations, followed by phenotypic screening to identify strains with reduced virulence. Examination of sufficiently attenuated strains has led to the identification of recurring genetic targets. For example, mutation of rpoS, a regulator of acid tolerance responses, is a defining feature of the Ty21a vaccine strain [18]. Other attenuation targets include genes involved in bile resistance, resistance to gastrointestinal defensins, and cell wall biosynthesis [17]. In practice, attenuation strategies often combine multiple mutations within a single strain, with varying effects on fitness and immunogenicity.

More recent attenuation approaches emphasize controlled or delayed attenuation phenotypes. These strategies allow vaccine vectors to initially retain near–wild-type characteristics following administration, followed by progressive attenuation that facilitates host-mediated clearance [19,20,21,22,23]. Delayed attenuation can be achieved through a range of mechanisms, including regulated metabolic auxotrophy or delayed expression of factors that impair bacterial fitness [24,25,26].

Achieving an appropriate balance between attenuation and invasiveness is critical for vaccine efficacy and is currently not fully understood or defined. Excessive attenuation can compromise survival through the gastric environment, translocation across the intestinal epithelium, and intracellular replication, all of which contribute to the induction of mucosal immune responses following oral delivery. Consequently, several groups have employed attenuated yet hyperinvasive *Salmonella* strains as vaccine vectors.

One such strain is *S.* Typhimurium YS1646, which is derived from the hyperinvasive strain YS72. YS72 carries deletions in purI and xyl, resulting in adenine auxotrophy and impaired utilization of D-xylose as an energy source, respectively [27]. YS1646 additionally contains a deletion in msbB, which reduces endotoxicity by preventing terminal myristylation of the lipid A domain of lipopolysaccharide. In a phase I clinical trial evaluating its use as a cancer therapeutic, 22 of 25 patients cleared the bacteria from the bloodstream within 12 h following intravenous administration, and no shedding was detected in urine or stool [28]. This strain also induced measurable systemic cytokine responses, including IL-1β, TNF-α, IL-6, and IL-12. Owing to its documented safety profile and invasive properties, YS1646 has since been repurposed as an orally delivered vaccine vector targeting mucosal pathogens such as *Clostridioides difficile*, *Schistosoma mansoni*, and *Cryptosporidium parvum*.

## 4. Immune Responses to Wild-Type *Salmonella* Typhimurium Infection

To appreciate the potential of *Salmonella* Typhimurium as a vaccine vector, it is necessary to understand the immune responses generated during wild-type infection, as these responses may resemble those elicited by live-attenuated vaccine strains. In the following sections, we discuss the innate, adaptive, and memory responses to wild-type *S.* Typhimurium infection, providing a framework within which vaccine-induced immunity can be examined. It is important to note that while *S.* Typhimurium infection in humans is largely restricted to the gastrointestinal tract, the organism disseminates systemically in mice, and immune responses differ depending on tissue context. The majority of our understanding of the immune responses to *S.* Typhimurium comes from studies in mice; however, this review will focus mainly on the responses described in the gastrointestinal tract.

### 4.1. Early Responses

**Figure 1:**
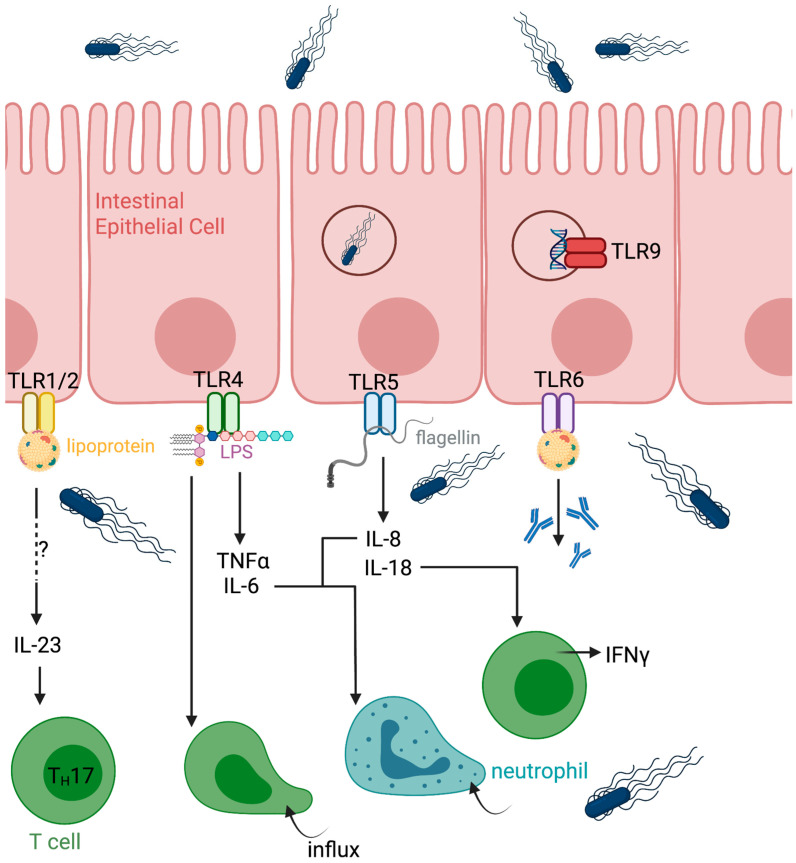
**Early events during *Salmonella* Typhimurium infection at the intestinal mucosa.** Following oral exposure, *S.* Typhimurium crosses the intestinal epithelium, preferentially through microfold cells overlying Peyer’s patches, and enters the lamina propria. Bacterial recognition by intestinal epithelial cells and resident immune cells through pattern recognition receptors initiates local inflammatory signaling. *S.* Typhimurium is subsequently phagocytosed by macrophages and dendritic cells, enabling intracellular survival and dissemination within mucosal tissues. These early events establish the inflammatory environment that shapes downstream innate and adaptive immune responses. Created in Biorender.

One of the advantages shared by *Salmonella*-vectored vaccines is their intrinsic “auto-adjuvanted” nature. As a pathogen, *S.* Typhimurium expresses multiple pathogen-associated molecular patterns (PAMPs) that are detected by both surface and intracellular pattern recognition receptors (PRRs). Toll-like receptors (TLRs) represent an early line of recognition during infection [29]. Upon reaching the lamina propria, *S.* Typhimurium encounters TLRs expressed on the basolateral surface of intestinal epithelial cells (IECs) (Figure 1). Several *S.* Typhimurium components activate TLR signaling, including lipoproteins (TLR1/2/6), lipopolysaccharide (TLR4), flagellin (TLR5), and proteins present in *Salmonella* biofilms that activate TLR2 [30,31]. CpG-rich motifs within *Salmonella* DNA further engage TLR9.

Activation of TLRs induces cytokine secretion by host cells, generating a pro-inflammatory environment within the gut mucosa. TLR4 signaling in IECs promotes secretion of TNFα and IL-6 (Figure 1) [32]. Flagellin-mediated TLR5 activation in humans induces IL-8 and IL-18 production [33]. IL-18 secretion is dependent on flagellin recognition [34] and serves as a potent stimulus for IFNγ production by mucosal-resident T cells [35]. TLR activation also leads to IL-23 production, although the precise cellular sources of IL-23 during *S.* Typhimurium infection remain incompletely defined [31]. Macrophages and dendritic cells produce IL-23 in vitro, while T cells, natural killer (NK) cells, and innate lymphoid cells (ILCs) are also potential contributors in vivo. IL-23 is required for IL-17 and IL-22 production by mucosal-resident T cells and promotes IL-22 secretion by ILC3s [36,37]. Notably, IL-22 production by ILC3s has been shown to impair bacterial clearance in murine models [38]. TLR4 signaling enhances CD4^+^ T-cell responses to *Salmonella*, whereas TLR5 signaling preferentially augments antibody responses [39]. TNFα, IL-6, and IL-8 further contribute to the recruitment of neutrophils and other inflammatory cells to the site of infection.

**Figure 2:**
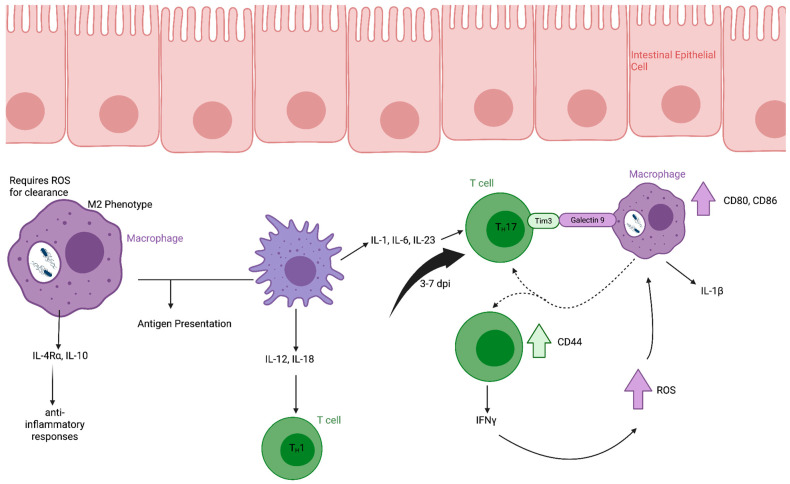
**Macrophage and CD4^+^ T cell responses to *Salmonella* Typhimurium infection in the lamina propria.** Following translocation across the intestinal epithelium, *S.* Typhimurium is phagocytosed by macrophages in the lamina propria. Actively infected macrophages can be polarized toward an M2 phenotype, characterized by secretion of IL-10 and expression of IL-4Rα, contributing to an anti-inflammatory environment. Reactive oxygen species production is required for intracellular bacterial clearance in infected macrophages. Both infected and uninfected macrophages participate in antigen presentation. Uninfected but activated macrophages produce cytokines that promote Th1 and Th17 differentiation of CD4^+^ T cells. Activated CD4^+^ T cells secrete IFNγ and IL-17, which enhance macrophage antimicrobial activity and contribute to bacterial control at the mucosal site. Created in Biorender.

As *S.* Typhimurium crosses the intestinal epithelial barrier and establishes a strongly inflammatory environment, bacterial cells are phagocytosed by macrophages and dendritic cells within the lamina propria [31]. Lamina propria macrophages are indispensable for controlling *S.* Typhimurium infection [40] (Figure 2). Reactive oxygen species (ROS) generated by infected phagocytes contribute to bacterial growth control [41,42]. However, *S.* Typhimurium can actively modulate macrophage polarization. Intracellular bacteria residing within the *Salmonella*-containing vacuole secrete SteE, which activates STAT3 and promotes an M2-like phenotype in infected macrophages [43]. M2-polarized macrophages are more permissive to *Salmonella* survival and are characterized by production of IL-10, expression of IL-4Rα, and anti-inflammatory properties. If unaddressed in vector design, this could lead to a dampened immune response to vaccine vectors.

Both infected and uninfected phagocytes can present antigen to naïve T cells within Peyer’s patches and shape downstream Th1- and Th17-skewed responses through cytokine-mediated crosstalk [44] (Figure 2). In contrast to infected macrophages, uninfected but activated macrophages produce IL-12 and IL-18, promoting IFNγ-dependent Th1 responses [45]. Production of IL-1, IL-6, and IL-23 further supports Th17 differentiation and recruitment of neutrophils to inflamed tissue [36].

Neutrophils play a critical role in bacterial killing within the intestinal lumen following their recruitment to the gut [46]. Their presence is essential for limiting bacterial dissemination [47,48,49], although excessive neutrophil accumulation can contribute to immunopathology through collateral damage to IECs.

Collectively, these early innate responses shape downstream adaptive immunity. Cytokine production by IECs, macrophages, and ILCs establishes a Th17- and Th1-skewed environment within the intestine. During infection, RORγt^+^ T-bet^+^ ILC populations in the colon become activated and migrate to the mesenteric lymph nodes, where they enhance IFNγ production and influence T-cell priming [50].

### 4.2. Adaptive Responses

CD4^+^ T cells play a central role in the clearance of *S.* Typhimurium infection [51]. T cell responses to infection vary depending on tissue location, reflecting both local immune environments and the distinct intracellular lifestyles adopted by *S.* Typhimurium in different tissues [52]. In murine models, the number of CD4^+^ T cells increases in the lamina propria (LP) between 3–7 days post-infection (dpi) [40]. Bidirectional crosstalk between CD4^+^ T cells and macrophages contributes to bacterial control. CD4^+^ T cells activate infected macrophages through TIM-3 and galectin-9 interaction, increasing CD80 and CD86 expression and promoting IL-1β production by the macrophage. While macrophages reciprocally enhance CD4^+^ T-cell activation and their CD44 expression and IFNγ secretion [40].

During intestinal infection, CD4^+^ T cells in the colonic LP are initially skewed toward a Th17 phenotype but progressively transition to a Th1 phenotype by approximately 11 dpi, characterized by T-bet expression and IFNγ production [53]. This transition is independent of bacterial persistence but requires the presence of regulatory T cells within the colonic LP. IFNγ production in the LP and mesenteric lymph nodes contributes to bacterial clearance by activating reactive oxygen species (ROS) production in macrophages [52]. *Salmonella*-specific CD4^+^ T cells persist within the LP for up to 90 days following infection in mice, highlighting the potential of *Salmonella*-vectored vaccines to induce durable mucosal T-cell immunity against gastrointestinal pathogens [53].

In mice, systemic adaptive responses differ from those observed at mucosal sites. In the spleen, myeloid-derived suppressor cells (CD11b^+^ Gr1^+^) are expanded during *S.* Typhimurium infection and serve as a bacterial reservoir [54]. These cells modulate T-cell responses by decreasing IL-2 production while enhancing IFNγ and IL-17 secretion through an iNOS-dependent pathway within five days of infection [54].

Evidence from large animal models further supports the relevance of these adaptive responses. In a porcine model of attenuated *S.* Typhimurium vaccination followed by wild-type challenge, antigen-specific CD4^+^ T-cell responses were most prominent in jejunal and ileal lamina propria lymphocytes [55]. Vaccinated animals exhibited increased frequencies of multifunctional CD4^+^ T cells producing TNFα, IL-17A, and IFNγ compared with unvaccinated controls, with the majority of cytokine-producing cells displaying an effector memory phenotype. Given the physiological similarities between porcine and human gastrointestinal immunity, these findings provide important translational insight into the potential of *S.* Typhimurium as a vaccine vector.

Humoral immune responses are also elicited following *Salmonella* infection. Anti-*Salmonella* antibodies are generated in mice, pigs, and humans and correlate with later protection against subsequent disease [56,57,58,59]. In murine models, B-cell responses to *S.* Typhimurium occur predominantly at extrafollicular sites in the spleen, with minimal germinal center formation [60]. Although Th1 responses are required for bacterial clearance, IL-12 production inhibits T follicular helper differentiation, thereby limiting germinal center development [61]. IgM production begins approximately four days post-infection and peaks within two weeks, while IgG responses are delayed but substantially more expansive, with IgG2c predominating in C57BL/6 mice [62].

Despite the absence of conventional germinal center reactions, *Salmonella*-specific antibody responses are highly diverse. Early in infection, only a small fraction of B cells produce detectable *Salmonella*-specific antibodies, reflecting low initial affinity despite a broad B-cell receptor repertoire. These responses occur independently of TLR2, TLR4, MyD88, and T-cell signaling. Notably, somatic hypermutation has been demonstrated at extrafollicular sites and within GC-like structures, challenging classical paradigms of humoral immunity [62]. Mechanistically, *Salmonella* outer membrane protein A (OmpA) can upregulate activation-induced cytidine deaminase expression and promote class-switch recombination in vitro [63], while co-engagement of TLR5 and the B-cell receptor by flagellin induces T-cell-independent class-switched antibody responses [64,65].

Beyond antibody production, B cells can function as competent antigen-presenting cells under appropriate conditions [66]. During murine *S.* Typhimurium infection, multiple B-cell subsets in the spleen become infected at both early and chronic stages [67]. These infected B cells can cross-process and present bacterial antigens via both cytosolic and vacuolar pathways and upregulate co-stimulatory molecules, including CD40, CD80, and CD86, as well as the inhibitory ligand PD-L1. This phenotype has led to the proposal that PD-L1 expression enables *Salmonella* to exploit B cells as a niche for persistence during chronic infection in mice. Prevention of this phenotype via attenuation will benefit vaccine vector design.

Collectively, adaptive immune responses to *S.* Typhimurium infection are characterized by tissue-specific CD4^+^ T-cell differentiation, unconventional humoral immunity, and durable antigen-specific memory. While intestinal CD4^+^ T cells transition from Th17- to Th1-skewed responses to support macrophage-mediated bacterial clearance, splenic B cells contribute to antigen presentation and antibody production in murine models. These features highlight the potential of *Salmonella*-vectored platforms to elicit long-lasting protective immunity at mucosal sites.

### 4.3. Memory Responses

Current understanding of long-term memory responses elicited by attenuated *S.* Typhimurium vaccine strains remains limited. One study examining long term immunity following vaccination with the *S.* Typhi strain Ty21a in humans reported that 1.5 years after vaccination, anti-LPS IgG titers in the blood were approximately twofold higher than those observed in unvaccinated controls [68]. Vaccinated individuals also exhibited increased frequencies of flagellin-responsive CD4^+^ and CD8^+^ T cells in peripheral blood, along with enhanced polyfunctionality among responding CD4^+^ T cells. In contrast, no significant differences were observed in serum anti-LPS IgA titers or in antigen-specific CD4^+^ and CD8^+^ T cell populations at the duodenal mucosa between vaccinated and unvaccinated individuals.

Insights into potential memory responses elicited by attenuated *S.* Typhimurium-based vaccine platforms can also be drawn from murine studies. Several groups have evaluated generalized modules of membrane antigens (GMMA) derived from *S.* Typhimurium. GMMA are outer membrane vesicles released by genetically modified bacteria and contain lipopolysaccharide, porins, and additional outer membrane antigens. Following a single GMMA immunization, durable B cell responses were detected in both the spleen and bone marrow of mice up to 203 days or 29 weeks after vaccination [69]. When administered as a two-dose regimen ten weeks apart with Alhydrogel as an adjuvant, GMMA induced anti-O-antigen IgG responses in serum and intestinal compartments that persisted for up to 28 weeks after the second dose [70]. No significant differences in IgA production were observed compared with control animals at this time point.

Collectively, these findings suggest that *S.* Typhimurium-based platforms can induce long-lived systemic humoral immunity, particularly IgG-dominated responses, with more variable effects on mucosal IgA. Evidence for sustained T cell memory is less well defined. In a study of young Malawian children following natural *S.* Typhimurium infection, the frequency of circulating antigen-responsive CD4^+^ T cells peaked approximately 13 months after infection before declining over time [56]. Although the young age of participants and the context of natural infection must be considered, these findings underscore limitations in extrapolating long-term memory outcomes from Ty21a vaccination studies to attenuated *S.* Typhimurium vaccine vectors.

More recent work has begun to address these gaps. Oral vaccination with attenuated *S.* Typhimurium vectors targeting *Clostridioides difficile* has been shown to confer protection against disease several months following immunization, supporting the capacity of these platforms to induce durable protective immunity against heterologous mucosal pathogens [71]. However, the immunological correlates underlying long-term memory, including the relative contributions of tissue resident T cells, circulating memory subsets, and long-lived plasma cells, remain incompletely defined. Further studies examining the durability and quality of memory responses induced by attenuated *S.* Typhimurium vaccines will be critical for optimizing their use as mucosal vaccine vectors.

The ability of *S.* Typhimurium to elicit both mucosal and systemic immune responses, without spreading systemically in humans, makes it an attractive candidate for a live-attenuated vaccine vector. Current research in this field is limited to preclinical research but covers a broad range of pathogens.

## 5. *Salmonella* as Vaccine Vectors for Infectious Diseases

As discussed above, *Salmonella* possesses several characteristics that make it a promising vaccine vector for infectious diseases. These include its ability to induce both mucosal and systemic immune responses, its preferential targeting of intestinal microfold cells overlying gut-associated lymphoid tissue, and its capacity to carry and express heterologous antigens. In addition, *Salmonella* expresses a diverse array of pathogen-associated molecular patterns that contribute to its intrinsic adjuvant properties and support the induction of antigen-specific immunity following vaccination.

### 5.1. Vaccine Vectors for Viral Pathogens

Viral pathogens typically require strong cellular immunity and, in some cases, mucosal antibody responses for protection, making the Th1-biased and mucosal immune profile induced by *Salmonella* Typhimurium particularly relevant. *Salmonella*-vectored vaccine platforms have been widely investigated for viral pathogens, particularly in contexts where induction of mucosal immunity is desirable. Zhi et al. (2021) developed an attenuated *S*. Typhimurium strain, KST0666, using radiation mutation technology to express the foot-and-mouth disease virus VP1 protein under a stress-inducible system [72]. Oral immunization resulted in increased fecal IgA levels and elevated serum IgG and IgM titers compared with animals receiving non-recombinant *S.* Typhimurium. Vaccinated mice also exhibited increased frequencies of activated CD8^+^ T cells and enhanced production of antigen-specific IFNγ, IL-5, and IL-17A, indicating the induction of both humoral and cellular immune responses. In addition, vaccinated animals were protected against *Salmonella* challenge, suggesting the potential for dual protection against both the viral pathogen and the vector.

The ability of *S.* Typhimurium vectors to elicit mucosal immunity has been explored in the context of HIV vaccine development, as HIV transmission primarily occurs at mucosal surfaces. HIV-1 Gag, a conserved structural antigen, has been extensively evaluated using recombinant *S.* Typhimurium platforms. Studies expressing HIV-1 Gag either intracellularly or as a secreted antigen have demonstrated expansion of CD4^+^ T cells and induction of Th1-associated cytokines, including IFNγ, IL-2, and TNFα, alongside Th2-associated cytokines such as IL-4 and IL-5 following oral immunization [73,74].

Influenza virus antigens have similarly been delivered using *Salmonella* vectors. Pei et al. (2015) developed an attenuated *S.* Typhimurium strain expressing hemagglutinin antigens from H1N1 and H5N1 influenza viruses [75]. Oral vaccination protected mice against lethal challenge and induced significant increases in serum IgG and mucosal IgA titers, as well as antigen-specific IFNγ-producing T cells. Hajam et al. (2017) generated *S.* Typhimurium vectors expressing influenza hemagglutinin and matrix protein 2 ectodomain and observed robust antibody and cytokine responses following multiple routes of administration, although intramuscular and intraperitoneal delivery provided superior protection compared with oral vaccination [76]. More recently, live attenuated *S.* Typhimurium has been evaluated as a vaccine vector for SARS-CoV-2 antigens in murine models, where vaccination induced antigen-specific cellular and humoral immune responses [77] (Table 1).

### 5.2. Vaccine Vectors for Bacterial Pathogens

Protection against many bacterial pathogens relies on combined Th1/Th17 responses and antibody-mediated immunity, aligning with the immune signatures generated by attenuated *Salmonella* Typhimurium vectors. The use of *Salmonella* vectors to target bacterial pathogens has also been investigated. Xin et al. (2009) reported the development of an attenuated *S.* Typhimurium strain expressing pneumococcal surface protein A, a highly immunogenic antigen from *Streptococcus pneumoniae* [78]. Oral immunization elicited antigen-specific mucosal IgA responses and a Th1-biased IgG isotype profile, and vaccinated animals were protected against challenge delivered via multiple routes, including intraperitoneal, intravenous, and intranasal exposure.

*S.* Typhimurium-vectored vaccines have also been evaluated for *Staphylococcus aureus*. Xu et al. (2018) demonstrated that delivery of SaESxA and SaESxB antigens through the SPI-1 type III secretion system induced serum IgG and mucosal IgA responses, as well as antigen-specific IFNγ- and IL-17A-producing splenocytes [79]. Although vaccination prolonged survival following challenge, complete protection was not achieved, highlighting the challenges associated with vaccine development against *S. aureus*.

Attenuated *S.* Typhimurium strain YS1646 has additionally been evaluated as a vaccine vector targeting *Clostridioides difficile*. Vaccination strategies incorporating oral delivery induced both systemic IgG and intestinal IgA responses and conferred protection against lethal challenge [71,80]. Protective immunity persisted for several months following vaccination, supporting the durability of immune responses elicited by *Salmonella*-based vectors (Table 2).

### 5.3. Vaccine Vectors for Parasitic Pathogens

Parasitic infections often require mixed cellular and humoral immune responses, and in some cases Th2-associated mechanisms, which can be supported through *Salmonella*-based vectors using multimodal or adjuvant strategies. *S.* Typhimurium vectors have been developed to target both protozoan and helminth parasites. For protozoan infections, attenuated *S.* Typhimurium expressing gp63 has been shown to induce protective immunity against leishmaniasis following oral immunization. In silico-guided antigen selection has further enabled the development of vectors capable of reducing parasite burden and limiting visceralization in models of *Leishmania major* and *Leishmania donovani* infection [81].

The application of *Salmonella*-vectored vaccines to helminth infections is particularly attractive given the size and complexity of these organisms. Pompa Mera et al. (2013) demonstrated that oral or intranasal immunization with *S.* Typhimurium SL3261 expressing a *Trichinella spiralis* antigen resulted in significant reductions in parasite burden and was associated with elevated IgG1 titers and IL-5 production [82]. The same vector strain has also been used to target *Echinococcus granulosus*, where oral immunization induced IgG1, IgG2a, and IgA responses along with IFNγ, IL-2, and IL-5 production [83].

Additional studies have employed *Salmonella* vectors to target cestode and trematode infections. Ding et al. (2013) developed a *S.* Typhimurium vector expressing the *Taenia solium* oncosphere antigen TSOL18, which elicited sustained antibody responses and increased CD4^+^ and CD8^+^ T cell frequencies in mice, with comparable antibody responses observed in pigs [84]. Attenuated *S.* Typhimurium strain VNP20009 has also been used to target *Schistosoma japonicum* and *Schistosoma mansoni* [85,86,87]. More recent work demonstrated that oral vaccination with attenuated *S.* Typhimurium expressing *Schistosoma mansoni* Cathepsin B induces robust systemic IgG responses in combination with strong intestinal IgA production and results in significant reductions in adult worm burden and egg deposition in mice [88]. Notably, this study employed chromosomal integration of the vaccine antigen rather than plasmid-based expression, addressing limitations associated with plasmid stability and antibiotic selection while maintaining protective efficacy. Together, these studies highlight the capacity of *S.* Typhimurium-vectored vaccines to induce protective immune responses against helminth parasites and underscore shared design principles across platforms, including the use of the nirB promoter system (Table 3).

## 6. Considerations of Salmonella as a Vaccine Vector

Perhaps the most important consideration when selecting *Salmonella* Typhimurium as a vaccine vector is the type of immune response it induces. As outlined throughout this review, immune responses to *S.* Typhimurium during wild-type infection or following vaccination with attenuated strains are predominantly Th1 biased, with contributions from early Th17 responses. These responses are largely generated at the gastrointestinal mucosa, with additional systemic immune activation. When selecting target pathogens for *Salmonella*-vectored vaccines, these immunological features must be carefully considered. For pathogens where Th2-skewed responses are required for protection or clearance, additional strategies such as multimodal vaccination or incorporation of Th2-skewing adjuvants may be necessary.

Safety is a central consideration in the development of live attenuated *Salmonella*-vectored vaccines, particularly for use in vulnerable populations. Clearance of wild-type *S.* Typhimurium infection relies heavily on neutrophil function and CD4^+^ T cell responses. Individuals with reduced CD4^+^ T cell counts may therefore have difficulty clearing a live attenuated vaccine strain, potentially resulting in adverse events such as systemic infection or prolonged bacterial persistence. In addition, immunosenescence is associated with impaired neutrophil function, which may alter vaccine-induced immune responses and increase the risk of adverse outcomes in elderly populations [89,90,91,92]. These considerations are particularly relevant for vaccines intended for immunocompromised individuals, older adults, or populations with a high prevalence of immunocompromising conditions such as HIV infection.

Bacterial shedding represents an additional safety concern for live attenuated *Salmonella* vaccines. Wild-type *S.* Typhimurium can establish chronic infection, and vaccine strains must therefore be sufficiently attenuated to ensure complete clearance. Transient shedding has been documented following Ty21a vaccination, with most shedding resolving within four days post-vaccination [93]. Careful attenuation strategies and monitoring of shedding will be essential components of clinical development. An important safeguard in early clinical trials is the use of vaccine strains that remain susceptible to clinically relevant antibiotics, allowing for rapid clearance of the vector if required.

Another limitation of *Salmonella*-vectored vaccines relates to oral vaccine efficacy in low- and middle-income countries. Reduced immunogenicity of oral vaccines has been documented in these settings and has been attributed to factors such as malnutrition, concurrent enteric infections, and environmental enteropathy. Helminth co-infection has also been shown to impair immune responses to oral vaccines [94]. In some cases, targeted interventions such as treatment of ascariasis with albendazole have improved antibody responses to live attenuated oral cholera vaccination [95]. Multimodal vaccination strategies incorporating multiple routes of administration may help address these challenges. Notably, Ty21a represents an exception among oral vaccines, as clinical trials conducted in several low- and middle-income countries have demonstrated induction of intestinal IgA responses and robust cellular immunity in school-age children. This may reflect the ability of *Salmonella* species to target microfold cells and rapidly access gut-associated lymphoid tissue. As additional *Salmonella*-vectored vaccines are developed, it will be important to determine whether similar immunogenicity can be achieved in high-risk populations.

While much of the mechanistic understanding of *S.* Typhimurium infection and vaccine vector immunology is derived from murine models, these studies provide important insight into host–pathogen interactions, antigen delivery, and immune programming that inform vector design. We acknowledge that *S*. Typhimurium exhibits distinct pathogenic behavior in humans compared with mice, and that translation of preclinical findings remains a major challenge. Nevertheless, murine models continue to serve as a foundational platform for evaluating attenuation strategies, antigen expression systems, and immune outcomes prior to clinical investigation.

Alternative bacterial platforms, including generally regarded as safe (GRAS) organisms such as *Lactococcus* and *Lactobacillus*, have also been explored for mucosal delivery of vaccine antigens in humans [96,97]. These vectors offer favorable safety profiles but typically exhibit limited invasiveness and reduced capacity for intracellular antigen delivery and innate immune activation. In contrast, attenuated *S.* Typhimurium uniquely combines mucosal targeting with infection of professional phagocytes and intrinsic adjuvant activity, features that continue to motivate its investigation as a vaccine vector despite translational challenges.

Advances in molecular genetics have provided new tools for the design of live attenuated *Salmonella* vaccine vectors. Historically, heterologous antigen expression has relied largely on plasmid-based systems. Although balanced lethal vector-host systems have been developed to maintain plasmids without antibiotic resistance markers, plasmids remain mobile genetic elements and may vary in copy number between bacterial cells. Such variability may pose challenges during large-scale vaccine manufacturing. In contrast, chromosomal integration of heterologous antigen genes provides a stable and antibiotic-susceptible platform. Potential limitations of chromosomal integration include lower gene copy number and technical challenges associated with the insertion of large genetic sequences. These limitations can be mitigated through the use of strong promoters and careful selection of chromosomal insertion sites that remain transcriptionally active. Systems inserting heterologous genes downstream of constitutively expressed loci, such as *glmS,* have been successfully developed in *Salmonella* vectors [98]. In addition, CRISPR-based genome editing approaches have been demonstrated in *Salmonella* and may further facilitate chromosomal integration of heterologous antigens [99]. The optimal choice between plasmid-based and chromosomally integrated expression is likely to depend on the nature of the antigen and the desired timing and magnitude of antigen delivery.

Pre-existing immunity to *Salmonella* represents another important consideration. Individuals with prior exposure to wild-type *S.* Typhimurium or prior vaccination with *Salmonella*-based vectors may mount immune responses that limit the effectiveness of subsequent vaccination. Similar concerns were initially raised for adenoviral-vectored vaccines. However, clinical experience during the COVID-19 pandemic demonstrated that booster doses of adenoviral vaccines can enhance immune responses to heterologous antigens [100]. In the context of *Salmonella* vaccines, priming with Ty21a followed by boosting with a heterologous antigen-expressing Ty21a strain has been shown to induce detectable T cell responses to the heterologous antigen in humans, although humoral responses were not observed [101]. These findings suggest that repeated exposure to *Salmonella* vectors may preferentially support cellular immunity. An additional potential benefit of vector-directed immunity is the possibility of dual protection against both the target pathogen and non-typhoidal *Salmonella*, which remains a significant cause of global morbidity and mortality [102]. Potential strategies to mitigate pre-existing vector immunity include heterologous prime–boost regimens, multimodal vaccination approaches, and the use of alternative *Salmonella* serotypes or engineered vectors, although these strategies require further clinical evaluation.

Manufacturing considerations also support the use of *Salmonella*-vectored vaccines. *Salmonella* can be produced at low cost, does not require host cells for replication, and has a short doubling time. Existing *Salmonella* vaccines, such as Ty21a, require storage at 2 to 8 degrees Celsius, simplifying cold chain requirements. Lyophilized attenuated *Salmonella* strains with chromosomally integrated antigens have demonstrated stability at room temperature for several months, supporting the feasibility of distribution in resource-limited settings [88]. Compared with vaccines requiring ultra-low temperature storage, these properties represent a substantial logistical advantage.

Finally, the potential role of *Salmonella*-vectored vaccines within multimodal vaccination strategies warrants consideration. This may be particularly relevant for respiratory pathogens, where oral *Salmonella*-based vaccination alone may be insufficient and combined or alternative routes may be required to achieve optimal protection. Oral delivery of *Salmonella* vectors may not consistently induce strong systemic IgG responses, which are commonly used as markers of vaccine immunogenicity. However, co-administration with parenteral recombinant antigen has been shown to enhance systemic antibody titers compared with parenteral vaccination alone. While multimodal approaches introduce additional complexity in safety testing and regulatory evaluation, recent regulatory flexibility surrounding heterologous vaccine schedules suggests that such strategies may be acceptable when supported by robust efficacy data. Together, these considerations highlight both the opportunities and challenges associated with the development of *Salmonella*-vectored vaccines and underscore the importance of careful platform design tailored to specific target populations and pathogens.

## 7. Conclusions

This review has discussed multiple aspects of *Salmonella* Typhimurium-vectored vaccines. A key advantage of these vaccines is their ability to target the gut mucosa. Targeting mucosal pathogens with mucosal vaccines enables the development of tissue-resident memory cells that can respond rapidly upon pathogen encounter. In addition to targeting mucosal pathogens, *Salmonella*-vectored vaccines have demonstrated versatility in their ability to induce immune responses against a broad range of infectious agents beyond the gastrointestinal tract. Innate immune cells responding to wild-type *S*. Typhimurium infection generate signals that promote IFNγ, IL-17, and IL-22 production by mucosal T cells. *Salmonella*-specific CD4^+^ T cells have been shown to persist in the lamina propria for up to 90 days in mice [53]. Although knowledge of B cell responses to infection remains incomplete, several studies have demonstrated that *S*. Typhimurium infection and vaccination can generate both IgG and IgA responses.

From these collective studies, several design principles emerge for *Salmonella*-based vaccine vectors, including the need to balance attenuation with epithelial translocation, to preserve intracellular antigen delivery while minimizing immune evasion, and to optimize antigen expression timing and stability. Effective vectors must retain sufficient invasiveness to access gut-associated lymphoid tissue while avoiding excessive systemic dissemination. In addition, vector engineering strategies that enhance antigen presentation while limiting anti-inflammatory polarization may improve heterologous immune responses. Together, these considerations highlight the importance of integrating bacterial genetics with host immunology when designing next-generation *Salmonella* vaccine platforms.

While the use of heterologous antigen-expressing *Salmonella* represents an older vaccine platform, advances in molecular genetics and mucosal immunology over the past two decades have renewed its relevance. These advances have enabled more precise control over antigen expression and attenuation strategies, supporting the development of safer and more effective vaccine candidates, particularly for mucosal pathogens. In this review, we have outlined the immune responses generated by *Salmonella* Typhimurium infection, summarized current *Salmonella*-vectored vaccine candidates, and discussed both the advantages and limitations of this platform. Together, these considerations highlight the continued potential of *Salmonella*-vectored vaccines and underscore the importance of addressing remaining gaps in knowledge to fully realize their utility.

## Figures and Tables

**Table 1:** *Salmonella*-vectored vaccines for viral diseases.

Target Pathogen	*Salmonella* Vector	Antigen	Route of Administration	Key Immune Responses	Protection	Reference
Foot-and-mouth disease virus (FMDV)	*S*. Typhimurium KST066	VP1	Oral	↑ IgA; ↑ IgG/IgM; ↑ CD8^+^ T cells; ↑ IFNγ	Yes	[72]
HIV-1	*S.* Typhimurium ΔaroC	Gag	Oral	↑ CD4^+^ T cells; ↑ IFNγ, IL-2, TNFα; ↑ IL-4, IL-5	Not assessed *	[73,74]
Influenza A (H1N1, H5N1)	*S.* Typhimurium ΔaroA (SL7207)	HA	Oral	↑ IgG; ↑ IgA; ↑ IFNγ	Yes	[75]
Influenza A	*S.* Typhimurium JOL1800	HA + M2e	Oral; IM; IP	↑ IgG1, IgG2a; ↑ IFNγ, IL-4, IL-10	Oral: decreased viral loads; IM & IP: Yes	[76]
SARS-CoV-2	*S.* Typhimurium Δasd (pVAX1)	Spike	Oral	↑ CD4^+^ + CD8^+^ T cells; ↑ IFNγ; ↑ IgG	Not assessed *	[77]

↑ indicates an increase. * Not assessed indicates that challenge experiments were not performed in the referenced study and that protection was inferred from immunological readouts.

**Table 2:** *Salmonella*-vectored vaccines for bacterial diseases.

Target Pathogen	*Salmonella* Vector	Antigen	Route of Administration	Key Immune Responses	Protection	Reference
*Streptococcus pneumoniae*	*S*. Typhimurium	PspA	Oral; IP; IV; IN	↑ mucosal IgA; ↑ Th1-skewed IgG isotype	Yes	[78]
*Staphylococcus aureus*	*S*. Typhimurium	SaESxA, SaESxB	Oral	↑ serum IgG; ↑ mucosal IgA; ↑ IFNγ, IL-17A	Yes	[79]
*Clostridioides difficile*	*S*. Typhimurium YS1646	RBD of toxins A and B	Oral; IM protein	↑ intestinal IgA; ↑ serum IgG	Yes	[71,80]

↑ indicates an increase.

**Table 3:** *Salmonella*-vectored vaccines for parasitic diseases.

Target Pathogen	*Salmonella* Vector	Antigen	Route of Administration	Key Immune Responses	Protection	Reference
*Leishmania* spp.	*S*. Typhimurium KST066	Gp63	Oral	↑ Th1 CD4^+^ T cells	Yes	[81]
*Trichinella spiralis*	*S*. Typhimurium SL3261	Ag30	Oral; IN	↑ IgG1; ↑ IL-5	Yes	[82]
*Echinococcus granulosus*	*S*. Typhimurium SL3261	EgDf1	Oral; IV	↑ IgG1, IgG2a; ↑ IgA; ↑ IFNγ, IL-2, IL-5	Not assessed *	[83]
*Taenia solium*	*S*. Typhimurium χ4558	TSOL18	Oral	↑ serum antibodies; ↑ CD4^+^ and CD8^+^ T cells	Yes	[84]
*Schistosoma japonicum*	*S*. Typhimurium VNP20009	Sj23LHD-GST	Oral; IM protein	↑ IgG2a; ↑ IL-12; ↑ IFNγ	Yes	[85]
*Schistosoma mansoni*	*S*. Typhimurium YS1646	CatB	Oral; IM protein	↑ serum IgG; ↑ intestinal IgA; ↑ IFNγ; ↑ IL-5	Yes	[86,87,88]

↑ indicates an increase. * Not assessed indicates that challenge experiments were not performed in the referenced study and that protection was inferred from immunological readouts.

## Data Availability

No new data were created or analyzed in this study. Data sharing is not applicable to this article.
